# Pilot study of telemedicine for the initial evaluation of general surgery patients in the clinic and hospitalized settings

**DOI:** 10.1016/j.sopen.2019.06.005

**Published:** 2019-07-12

**Authors:** Caleb Schroeder

**Affiliations:** Mary Lanning Healthcare, 715 N Kansas Ave., Suite 205, Hastings, NE, 68901

## Abstract

**Background:**

Telemedicine has had limited implementation for general surgery. The purpose of this study was to evaluate telemedicine for the initial evaluation of patients in the clinic and hospital settings.

**Methods:**

Synchronous telemedicine consults were conducted by a single surgeon to a rural hospital and clinic. Reasons for consult, adequacy of consult, days saved by telemedicine consult compared to standard practice, correlation of telemedicine and in-person physical exam, and number of patients who required procedures were evaluated.

**Results:**

On average, patients were evaluated 7.4 days more rapidly than if the consult had been done by our standard practice. Telemedicine was adequate for all patients in this study.

**Conclusions:**

This is the first study using telemedicine for the initial consult of general surgery patients in the hospitalized and clinic setting in North America. The physical exam remains an important component of the general surgery evaluation, and special attention must be considered when structuring the telemedicine program. Telemedicine is an effective and expedient way to provide consultation for general surgery patients. Further study is needed to determine which general surgery issues are not amendable to telemedicine consultation, and to determine other surgical specialties that could utilize telemedicine in their practice.

## Highlights

• This is the first study using telemedicine for general surgery in the United States.
• Telemedicine has potential for use in general surgery in both the clinic and hospitalized settings.
• Regulatory restraints on telemedicine are decreasing nationally.
• The physical exam requires special attention for the implementation of telemedicine for general surgery.
• Further study is required to determine which general surgery issues are amendable to telemedicine consults.

## INTRODUCTION

Telemedicine is gathering increased attention as a means to provide care in an expedient and cost-effective manner. It has been successfully implemented for pediatric care [1], stroke care [2], diabetes management [3], and intensive care management [4]. There is also increased interest in implementation for pediatric surgery [5] in the United States, and internationally it has been implemented with excellent success [6].

Telemedicine has had limited implementation for general surgery. It has been utilized for postoperative visits [7] and breast care [8]. Guidelines have not been created for the use of telemedicine in general surgery [5]. The only published study utilizing telemedicine for general surgery consultation in a clinic was conducted by Cain et al. [9]. Their study was completed by the United States Military stationed in Europe. Six centers throughout Europe conducted telemedicine with surgeons at a central location. Commonly encountered general surgery problems such as hernias, lipomas, gallbladder complaints, and evaluation for endoscopy were examined. Authors concluded that this method “was safe and effective in evaluating common general surgical conditions”. There are no published studies utilizing telemedicine for general surgery consultation of hospitalized patients.

Telemedicine has special interest for the care of patients in rural settings. These patients compromise a significant portion of the population in the United States and across the world. The potential to provide specialist evaluations to these patients often requires extensive travel by patients or providers. Nebraska is a state in the United States with a large rural population, and several recent local and national developments have provided the opportunity for the advancement of telemedicine. Nebraska state legislature recently passes a law which requires insurance providers to refuse to deny coverage for the sole reason that the care was given via telemedicine. Nebraska also has existing telemedicine equipment in place in rural and urban hospitals through a grant for the Nebraska Statewide Telehealth Network (NSTN). Through the NSTN, over 110 sites in the state have virtual instant access to other sites in the NSTN. Several other states have similar telehealth networks. Passage of the Bipartisan Budget Act of 2018 also has produced increased insurance coverage for telehealth for Medicare recipients.

Cain and colleagues demonstrated that telemedicine could be completed for general surgery patients, but their study was completed in the setting of the Armed Forces stationed in Europe. There have been no studies to implement telemedicine for general surgery patients in the continental United States. The purpose of our study was to determine if general surgery patients could be effectively evaluated through the use of telemedicine.

## METHODS

This study was conducted at Brodstone Hospital (originating site) in Superior, Nebraska and Mary Lanning Hospital (distant site) in Hastings, Nebraska. These towns are located approximately 60 miles apart and have populations of 1856 and 24,991, respectively. Both are located in rural areas and draw from a substantial population in surrounding communities. This study involved a single surgeon located at the distant site. Our typical practice was to travel to the originating site every 2 weeks to see consults and perform outpatient surgery.

Our population for this study consisted of all patients seen at the originating site who were referred by their primary care provider to require surgical evaluation amendable to telemedicine consultation. Referrals were made at the discretion of the provider at the originating site, taking into account the acuteness of the patient, appropriateness of the surgical issue, and patient preference. Patients were given the option of an in-person interview either by travel to the distant site or by waiting until scheduled evaluation at outreach clinic. All patients provided written informed consent for the telemedicine consult. This study was started in March of 2018 and data was reviewed in December of 2018. This population included clinic patients and patients who were admitted to the hospital. Patients were evaluated with the assistance of the NSTN or Omnijoin software. Interviews were conducted using 64 inch live action monitors, with HD video devices mounted at the top of the device. Users were able to remotely move the camera, and remotely zoom the camera as needed. All devices came with a two way speaker and receiver. All communication was conducted using a secure, HIPAA compliant connection. Evaluation consisted of synchronous evaluation between surgeon and patient. The provider was located in his office at the distant site, patients were located either in the specialty clinic or in their hospital rooms at the originating site. Physical exam was completed with the assistance of a physician, physician assistant, or nurse practitioner at the originating site. Physical exam focused on examination of the heart, lungs, and abdomen, and was reported verbally by the preforming physician as each portion was completed. A nurse was present at all times at the originating site to ensure appropriate function of equipment. All patients underwent physical exam by the surgeon prior to surgery, or at their next clinic visit if surgery was not scheduled. For all patients, the office at the distant site obtained labs and radiology reports for review at the time of the telemedicine consultation. Images were electronically transferred from the originating site to the distant site, and were personally reviewed by the surgeon at the distant site at the time of the telemedicine consultation.

Institutional oversight was completed through discussion with all physicians and administration at the originating site, and through discussion with physician leaders and administration at the distant site. These persons functioned as an equivalent of institutional review boards at our rural institutes. These discussions with administrative and physician leaders occurred before any patient interactions through telemedicine.

Information tracked included patient gender, age, setting of evaluation (clinic vs. inpatient), reason for consult, days saved before evaluation by standard means, adequacy of telemedicine consult, correlation of physical exam by provider at originating site with in-person exam by surgeon, instances of surgery offered for the patient, days saved to surgery if applicable. For inpatient consults, we tracked if the consult would have happened without a telemedicine consult, decision to transfer patient as a result of consult, and number of serial evaluations that occurred. This is similar to methods employed by Cain et al. Due to the low number of cases, statistical analysis could not be meaningfully employed.

## RESULTS

Nine patients were evaluated from May to December of 2018. Results are listed in Table 1. There were 3 men and 6 women. Patient age range from 30 to 86. Six were located in the clinic, 3 were inpatients. Reasons for consult included gallbladder pain (2), small bowel obstruction (2), umbilical hernia, dysphagia, blood in stool, diarrhea, and weight loss. Days saved before evaluation by standard means were 59 total days for 9 patients, with average saving of 7.4 days. Procedures conducted as a result of this study include laparoscopic cholecystectomy (2), umbilical hernia repair, esophagoscope-gastro-duodenoscopy, and colonoscopy (3). For all patients, the time saved for operation was 14 days. Telemedicine was deemed to be adequate for all referrals. No patients required additional testing as a result of the interview. For patients with surgical issues meeting criteria for this study, 2 elected for in-person evaluation rather than telemedicine evaluation. These were both patients who were not hospitalized, and traveled to the distant site for clinic evaluation. Results for hospitalized patients are listed in Table 2. One of the patients would have been evaluated in person by the surgeon without the use of telemedicine, two would not have been evaluated as they were admitted and discharged in the two weeks that the surgeon was not present at the originating site. Evaluation by the surgeon would have required transfer to the distant site. None of the patients required transfer. One patient required serial evaluation.

**Table 1:** Patient demographics and outcomes

Patient	Setting	Reason for consult	Days before evaluation without TM	Adequate evaluation	Correlation to in-person exam	Procedure offered
1	Clinic	Gallbladder pain	6	Yes	Yes	Yes
2	Inpatient	SBO	12	Yes	N/a	No
3	Clinic	Umbilical hernia	12	Yes	Yes	Yes
4	Clinic	Gallbladder pain	7	Yes	Yes	Yes
5	Clinic	Dysphagia	5	Yes	Yes	Yes
6	Inpatient	Blood in stool	1	Yes	Yes	Yes
7	Clinic	Diarrhea	6	Yes	Yes	Yes
8	Clinic	Weight loss	6	Yes	Yes	Yes
9	Inpatient	SBO	4	Yes	N/a	No

**Table 2:** Outcomes for hospitalized patients

Patient	Evaluated without TM?	Decision to transfer	Serial evaluation
2	No	No	1
6	Yes	No	0
9	No	No	0

## DISCUSSION

This is the first study in the United States to employ telemedicine as a means for the initial evaluation of general surgery patients in the clinic and hospitalized setting. It is also the first study to use telemedicine for general surgery consultation of serial evaluation of hospitalized patients. Our study shows that telemedicine for general surgery patients can adequately determine the need for surgical intervention. None of the patients required transfer, but results would suggest that telemedicine could be an effective tool in stratifying those patients requiring transfer and those which may remain at the originating site.

This study confirms the potential for telemedicine to transform the way in which care is provided for general surgery patients in rural areas. This also has potential to expedite evaluations for urban centers in which surgeons cover multiple hospitals, or in which specialist evaluation is required. No patients were evaluated in the emergency department, but such evaluations would certainly be amendable to telemedicine consultation, and could significantly assist with determination for transfer as needed.

There are several opportunities for additional application of telemedicine as a direct result of this study. First, this study may be used as a template for a multi-center trial for the use of telemedicine in rural locations. There is simply not a large enough population in a single rural community to produce a study that will definitively address the use of telemedicine for general surgery. However, grouping of this data from multiple rural sites will provide this information. This study will also serve as a template for studies focused on the application of telemedicine at urban sites, for providers who practice at multiple locations. The critical piece of application for these sites is to determine the breadth of general surgery issues in which telemedicine can be utilized for the initial consult of the patient, and to identify the range of general surgery problems to which it can be applied. As part of larger studies, the use of telemedicine for postoperative care and remote monitoring could also be an exciting and meaningful application of this study. Postoperative follow up would be very beneficial, as patients in rural locations often travel long distances for an appointment that is typically short in duration. Remote monitoring could also provide the opportunity for urgent alterations in plans for general surgery patients, and potentially decrease the rate of unneeded interhospital transfers.

When preparing to conduct this study, special attention was given to the method by which we would complete the physical exam. It is the opinion of this author that a detailed physical exam by the preforming surgeon is an essential and irreplaceable part of the preoperative evaluation. Indeed, this may be a major contributing factor to the delayed implementation of telemedicine for general surgery patients. However, completion of the history by telemedicine, and completion of the physical exam by a competent telepresenter was felt to be adequate to make the decision regarding the need for surgery in this population. The correlation of physical exam by the telepresenter correlated completely with the in-person exam by the surgeon, on the day of surgery. This is likely because we utilized physicians, physician assistants, and/or nurse practitioners at the originating site for the physical exam. Other studies have used telepresenters consisting of nurses or medical technicians, further study is needed to determine if these team members would be appropriate for this critical aspect of evaluation of general surgery patients.

All hospitalized patients were evaluated on the date of request for evaluation. Specialist evaluation and recommendations urgently at critical care hospitals have significant potential to prevent needless transfers, assist the physician at the critical care hospital with initial management, and provide a significant savings in resources.

The patients evaluated in this study had a high rate of undergoing procedural intervention (78%). This is similar to the Cain study, in which telemedicine consultation resulted in 88% surgical intervention rate. We agree with Cain's conclusion that this was likely due to patients having a more thorough evaluation (imaging, etc.) by the primary care providers prior to consultation, and careful selection of patients deemed appropriate for evaluation by telemedicine.

There are several limitations to our study. Due to the low population in this rural location, the number of patients enrolled in this study is small. However, we believe that the results of this study will be easily applicable to other rural populations. It is unclear which patients, and what surgical issues, were not referred for telemedicine consultation by the primary care physicians. While telemedicine has a role to expedite care for the majority of general surgery issues, it may not be adequate for all general surgery issues and further study is required to determine cases that are adequate for telemedicine.

In conclusion, telemedicine is effective for the initial evaluation of general surgery patients. It is useful for the initial preoperative evaluation in clinic, and for the initial and serial evaluation of hospitalized patients. All patients should undergo in-person physical exam by the surgeon prior to operation, but determination for surgery can be completed based on the telemedicine consult. Further study is required to determine which general surgery issues are amendable to telemedicine consults and those that require in-person evaluation.

This research did not receive any specific grant from funding agencies in the public, commercial, or not-for-profit sectors.

## Author Contribution

Dr. Schroeder was responsible for all contributions related to this article.

## Conflict of interest

The author has no conflict of interest to report.

## Funding sources

None.
